# Experimental Validation of LiDAR Sensors Used in Vehicular Applications by Using a Mobile Platform for Distance and Speed Measurements

**DOI:** 10.3390/s21238147

**Published:** 2021-12-06

**Authors:** Ionuț Vasile, Emil Tudor, Ion-Cătălin Sburlan, Marius-Alin Gheți, Gabriel Popa

**Affiliations:** 1 National Institute for Research and Development in Electrical Engineering, Department of Renewable Energy Sources and Energy Efficiency, 313 Splaiul Unirii, 030138 Bucharest, Romania; ion.sburlan@icpe-ca.ro; 2 Department of Railway Vehicles, University POLITEHNICA of Bucharest, 313 Splaiul Independenței, 060042 Bucharest, Romania; gheti_marius@yahoo.co.uk (M.-A.G.); gabi21popa@yahoo.com (G.P.)

**Keywords:** LiDAR sensor, distance sensor, relative speed sensor, vehicle dynamics, control strategies, automated driving and autonomous vehicles, safety distance

## Abstract

LiDAR sensors are needed for use in vehicular applications, particularly due to their good behavior in low-light environments, as they represent a possible solution for the safety systems of vehicles that have a long braking distance, such as trams. The testing of long-range LiDAR dynamic responses is very important for vehicle applications because of the presence of difficult operation conditions, such as different weather conditions or fake targets between the sensor and the tracked vehicle. The goal of the authors in this paper was to develop an experimental model for indoor testing, using a scaled vehicle that can measure the distances and the speeds relative to a fixed or a moving obstacle. This model, containing a LiDAR sensor, was developed to operate at variable speeds, at which the software functions were validated by repeated tests. Once the software procedures are validated, they can be applied on the full-scale model. The findings of this research include the validation of the frontal distance and relative speed measurement methodology, in addition to the validation of the independence of the measurements to the color of the obstacle and to the ambient light.

## 1. Introduction

### 1.1. Distance Measurement Methods

Determining the distance to an object, its size and shape is a practical consideration for many current technical applications, such as remote detection, the counting of objects on a conveyor belt, equipment used for printed circuit board manufacturing, topological map creation or electron microscopy.

The literature [1] presents and analyzes the methods of determining distance using optical means. In Table 1, the main methods of determining distance are presented so we can observe the variety of these methods, each of which in turn has different peculiarities depending on the current application.

Regarding the solutions that can be applied in the vehicle safety applications, as Autonomous Emergency Braking Systems (AEB), the LiDAR is highly recommended for long-range distance measurements [2]. The authors of [3,4] propose various systems using neural networks or multiple LiDAR units to detect the presence of pedestrians. The authors of [5] present a method for very precise distance measurement using LASER rangefinders that has an uncertainty at the level of of 1 cm. The authors of [6,7] propose different methods for detecting obstacles using LiDAR sensors with applications in 3D measurements and autonomous guidance systems. From these references, we can conclude that precise distance measurement is of great importance especially for the implementation of safety systems.

In terms of the measurement of distance, there are two broad categories of techniques [8]:

- Methods using the principles of optics;
- Non-optical methods (sonar, capacitive, inductive).

Optical methods are divided into:

- Passive methods—in which the detection system does not illuminate the target object, this being achieved by ambient light or by the target object itself;
- Active methods—in which the detection system also emits light radiation; depending on the method used, it may be a combination of monochromatic, continuous, pulse-like, coherent or polarized radiation.

**Figure 1:**
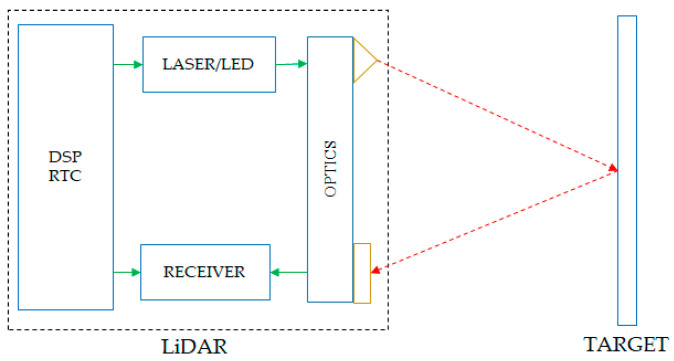
The basic diagram of a LiDAR sensor with time-of-flight measurement [8].

Both passive and active methods can be divided into three categories according to the measurement principle [8]:

- Interferometry—this method uses the wave aspect of light radiation and the fact that these waves can interfere with each other;
- Geometric methods—one example of this type of method is geometric triangulation, which is based on spatial relationships between the source, the target object and the detector sensor;
- Methods based on time measurement—these methods are based on the fact that the speed of light has a constant and finite value depending on the environment through which it propagates. This method is based on measuring the actual time elapsed from the light pulse emission until the radiation is received by the sensor. The diagram in Figure 1 shows the general principle of operation of this method:

In this case, the measured distance will have a value given by Equation (1):

d = c∙T/2(1)

where c is the speed of light, and T is the total time of flight of the radiation [5].

The LiDAR has a high-precision real-time counter (RTC) that measures the time it takes for a pulse of light to travel from the emitter, a laser or LED, to the target object and back to the LiDAR receiver. To ensure proper measurement, both the emitter and receiver are synchronized by this RTC.

**Figure 2:**
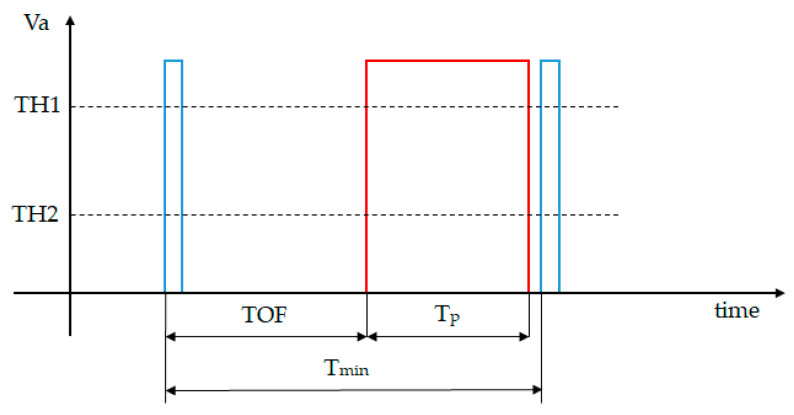
Synchronization of START and STOP pulses for time-of-flight [8].

In Figure 2, it is shown that there is a light pulse frequency limit, which is derived from the minimum time between two consecutive laser pulses, Pulse 1 and Pulse 2.

The operating frequency has the following formula:

Fmin = 1/Tmin(2)

where Tmin is expressed as the sum of the travel time with the accumulation/processing time by the receiver:

Tmin = TOF + Tp(3)

where TOF represents the duration of the time from the emission until the light is reflected and received and Tp is the time needed to process a sufficient amount of illumination so that the signal can be validated. The value of the processing time is set by a parameter that sets a threshold value, TH1 or TH2, that directly influences Tp.

### 1.2. LiDAR Sensors Operation Principle

**Figure 3:**
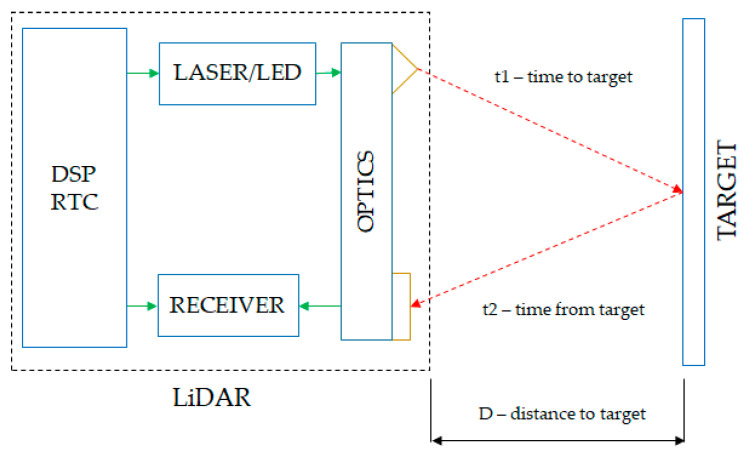
LiDAR measuring distance using time-of-flight [8].

A LiDAR sensor measures the travel time of a light pulse emitted from a source to a target object and back to a receiver, as seen in Figure 3. Laser-using systems belong to a category known as scanner-less LiDAR, where the entire object is swept with the pulse of light, in contrast to systems in which the object is scanned point-by-point [9].

The components of such a system are [8]:

- Lighting unit—this illuminates the scanned object with a pulse of light generated by a LASER or LED;
- Optical system—a lens accumulates reflected light and projects it onto the detector sensor;
- Image sensor—the main component of the system; a large majority of image sensors are composed of semiconductor materials (photodiode, CCD, MOS);
- Control electronics—has the role of synchronizing the emitter and the receiver in order to obtain the correct results;
- User interface—responsible for reporting the measurements over an external interface such as USB, CAN or Ethernet connection.

The simplest version of the LiDAR sensor that measures the time-of-flight uses pulses of light [10]. Thus, a short light pulse is emitted from a laser or LED; the resulting pulse illuminates the environment and is reflected by objects in the field of vision. The camera lenses inside the sensor receive this reflected light and focus it on the image sensor. Due to the fact that the speed of light is constant, it is possible to use Equation (1) to determine the distance to objects.

For example, for an object located at a distance of 1 meter, the time of flight is:

t = (2∙1)/(3∙108) = 6.7 ns(4)

The active duration of the pulse of light determines the maximum distance measured by the sensor. For a pulse with an active duration of 100 ns, the maximum measurable distance will be:

d = (3·108·100∙10^−9^)/2 = 15 m (5)

These results demonstrate the importance of the light source, resulting in the need to use special LEDs or a LASER capable of generating such pulses. 

The advantages of LiDAR sensors include [8]:

- Simplicity—LiDAR is compact and the lighting unit is placed next to the lens, thus reducing the size of the system;
- Efficient algorithm—distance information is extracted directly from the measurement of the flight time;
- Speed—such sensors are able to measure distances to objects in a certain area in a single light pulse sweep.
- The disadvantages of LiDAR sensors are as follows:
- Background light—can interfere with the normal functioning of the sensor, generating false results.
- Interference—if multiple cameras are used at the same time, they can interfere with each other, with both generating erroneous results.

### 1.3. LiDAR Sensor Vehicle Safety Application

It is important to introduce systems that can be used to manage the safety and security of the tram operations, which will identify and manage inherent events that appear during circulation. In this way, it will be possible to prevent accidents and improve risk control.

The tram represents a “light rail vehicle” that generally uses infrastructure with equipment that operates at speeds lower than those used in rail or subway networks. The integration of the tram in a multimodal system of public transport systems involves the strict compliance of the driving systems with safety and security regulations.

Many of the risks that the tram system manages are similar to those of railways or subways. However, there are some notable differences that introduce special risks.

The most significant difference is the operating principle. Unlike the rail or subway network where train movements are regulated by signaling systems, tram driving is conducted to the extent that the driving lane is free, with trams being driven “on sight” in a similar way to road vehicles. This offers greater operational flexibility that allows, for example, trams to travel closer to one another, while ensuring the stopping capacity of the vehicle. Tram drivers must operate the tram at a speed that allows them to stop the tram at a safe distance that they can clearly see. This is the same as the operating principle that is applied by the drivers of road vehicles, and it allows trams to operate near pedestrians and road vehicles [11]. For this reason, similarly to the railway system, many of the safety systems used in road vehicles have to be designed and adapted for trams according to the specific traffic network.

While the tram may have many similarities to buses and coaches, a fundamental element that differentiates them is the inability of the tram to reorient itself around an obstruction (such as reaching the rear of another tram, or the obstruction generated by a car or pedestrian), namely obstacles that suddenly enter in the tramway. This inability of the tram to avoid the obstacles, which is associated with the need for an increased braking distance, determines the need for a detection system that allows the driver to stop the tram in a timely manner in order to avoid an accident with very serious effects (considering the large inertial mass of the tram [12,13]).

The safety distance of a tram is its maximum braking distance, which is computed based on its actual speed. In cases in which another tram is rolling in front of it, the safety distance computing method must use both trams’ speeds. The tram must have a dedicated sensor to measure its own speed and the relative speed of the two trams in order to compute the safety distance and to warn the driver if the distance between the two trams has become smaller than the safety distance, indicating that a collision is possible.

The method involving the use of the safety distance requires only a LiDAR sensor for determination of the relative speed and distance to the object in front. The minimum braking distance can be directly computed according to the following formula:

d_min_ = v^2^/(2·a) (6)

where d_min_ is the minimum braking distance in meters, v is the tramcar’s actual speed in m/s and a is the maximum braking deceleration in m/s^2^. The maximum braking deceleration is imposed for trams. Knowing the relative speed between the two trams, v_r_, and the actual speed of the LiDAR tram, v_1_, the speed of the front tram can be computed with the following formula:

v_2_ = v_1_ − v_r_ (7)

Knowing v_2_, we compute the minimum braking distance of the front tram using the same formula (6). The threshold distance, d_th_, at which a warning signal is generated, is calculated with the formula:

d_th_ = d_1_ − d_2_ (8)

where d_1_ and d_2_ are the braking distances of the two trams.

When the distance obtained from the LiDAR sensor is smaller than the threshold distance, the system signals to the driver that a collision is possible.

Other methods, such as time-to-collision, require a large selection of sensors such as radar, LiDAR, a camera for image recognition and GPS for localization, and all these additional sensors require more complex hardware and software procedures to determine the time of collision [14].

**Figure 4:**
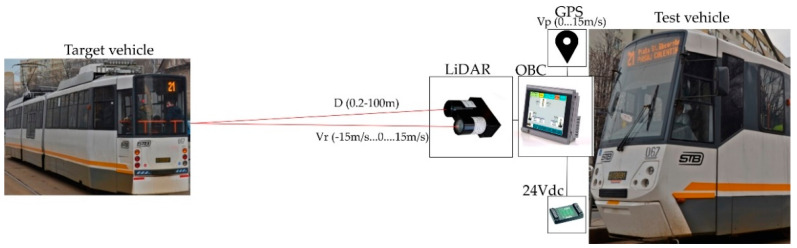
Configuration of LiDAR system used for tramcar safety.

Currently, the trams are not equipped with obstacle detection systems, which make them vulnerable in terms of preventing and avoiding accidents. A possible configuration for a tramcar LiDAR safety system is presented in Figure 4. In this setup, a tram is equipped with a long-range and narrow FOV LiDAR sensor, a GPS system, an onboard computer system/display (OBC) and a power supply (24Vdc).

The operation of the trams may involve an increased level of interaction with the trams running in front, as well as the road vehicles and pedestrians, as compared to railway operations. This should be managed to prevent and mitigate the effects of collisions. Although, compared to the railway vehicles, the levels of kinetic energy and of the forces in collision events are lower (determined by lighter load structures and lower movement speeds), they remain extremely high if there is a collision with another tram, car or pedestrian.

In the absence of specific legislation regarding the safety and security of the tram systems and associated infrastructure, manufacturers and operating companies are interested in developing and implementing traffic control systems for the prevention of collision events in order to ensure a high level of safety and security [15].

As in the case of road vehicles, the main risk is the tram driver, their reaction time and the reduced event prevention capacity determined by the “on sight” operating mode.

There is a need for a technology to monitor and support the tram driving activity (a rapidly developing area today) in order to increase the reliability of the operation and increase the “on sight” observation capability in order to reduce the risk exposure.

**Figure 5:**
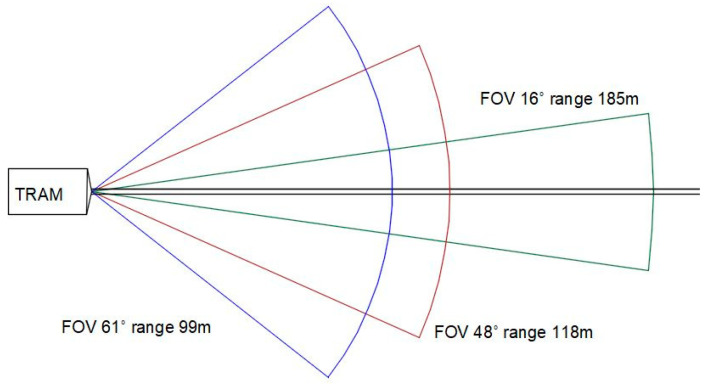
FOV angle and range for LiDAR sensors in vehicle autonomous driving [16].

Vehicles of all types use LiDAR to determine which obstacles are nearby and how far away they are. The 3D maps provided by LiDAR components are used not only to detect, but also to position objects and also identify what they are, using complementary video cameras, as in Figure 5. Insights uncovered by LiDAR also help a vehicle’s computer system to predict how objects will behave, and thus, to adjust the vehicle’s driving accordingly.

Semi- and fully-autonomous rail vehicles use a combination of sensor technologies [17,18]. This sensor suite includes a microwave RADAR or a LiDAR, which provides constant distance and velocity measurements as well as superior all-weather performance, but lacks in resolution, and struggles with the mapping of finer details at longer ranges [19]. Camera vision, also commonly used in automotive and mobility applications, provides high-resolution information in 2D. However, there is a strong dependency on powerful Artificial Intelligence and corresponding software to translate the captured data into 3D interpretations [20]. Environmental and lighting conditions may significantly impact camera vision technology [21,22].

LiDAR, in contrast to video cameras, offers precise 3D measurement data over short to long ranges, even in challenging weather and lighting conditions. This technology can be combined with other sensory data to provide a more reliable representation of both static and moving objects in the vehicle’s environment.

Hence, LiDAR technology has become a highly accessible solution to enable obstacle detection and avoidance, and safe navigation through various environments, in a variety of vehicles. Today, LiDAR is used in many critical automotive and mobility applications, including Advanced Driver Assistance Systems and autonomous driving (Table 2).

The sensors that are available on the market are dedicated to several applications, for example:

- Short-range sensors are dedicated to virtual machinery;
- Medium-range sensors have been developed for small robots and automated tools and long-range sensors are used for safety tools and perimeter alarms;
- Wide-range sensors have been built for safety tools for use in automated machinery;
- Three-hundred-and-sixty degree sensors are used for automated vehicle driving.

Similar works are presented in the [23], which is focused on the avoidance of static obstacles by means of shape recognition at a short distance. The influence of road conditions is analyzed in [24], where LiDAR performances are presented under various weather conditions.

In a recent review [25], the Frontal Collision Warning, FCW and Autonomous Emergency Braking (AEB) systems were evaluated to be implemented using both LiDAR and long-range RADAR by monitoring time to collision (our methodology uses distance measurement).

## 2. Materials and Methods

### 2.1. Test Platform Hardware

**Figure 6:**
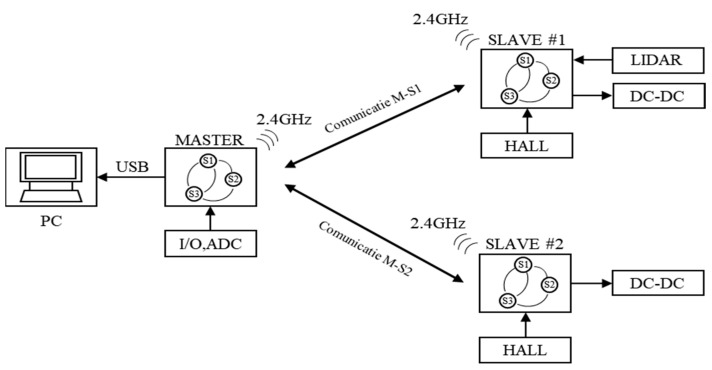
Basic schematic of the LiDAR testing platform.

The experimental model we designed for indoor tests has the basic schematic presented in Figure 6, and consists of a master control unit connected to a local computer that communicates by radio with two mobile trains with independent speed control.

Each train is equipped with an onboard converter that controls the speed, a radio receiver to monitor the command from the master unit, and a hall sensor used to determine the absolute speed of the train, and one of the trains is also equipped with a LiDAR sensor for distance measurements. Each unit is equipped with a processor that handles all necessary software functions.

#### 2.1.1. Master Control Unit for Remote Control and PC Interface

The master control unit (MCU) was used to send commands to two mobile trains—one that was equipped with a LiDAR sensor and one that acted as a target vehicle. The MCU was also responsible for sending data to a local computer for logging purposes. The communication between the MCU and the secondary mobile modules was implemented by a radio communication network in the 2.4 GHz frequency range.

**Figure 7:**
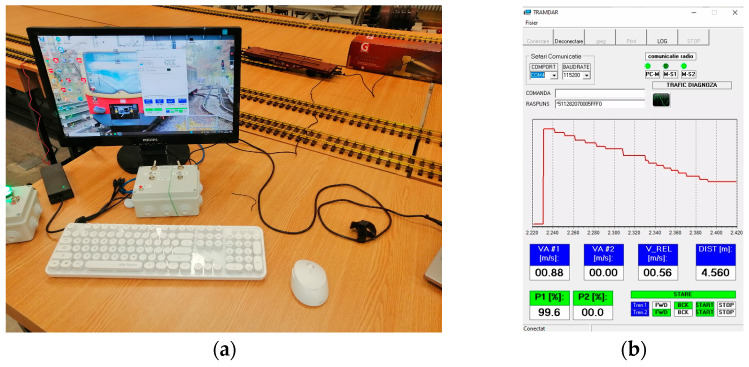
Master Control Unit setup: (**a**) data analyzed on the PC; (**b**) online data analyzed on the PC (screen capture).

The MCU (see the white box from Figure 7) consisted of an ATmega2560 microprocessor [26], connected with 4 digital switches that controlled the type of motion of the two secondary units and with two analog potentiometers that controlled the duty cycle of each DC–DC converter. More specifically, each secondary module could be controlled remotely and independently with variable duty cycles of the on-board DC–DC converter to obtain a desired speed and with forward/stop/backward direction of movement. The MCU acted as a double remote controller.

To implement the radio network module, the nRF24L01 chip was used [27], as it has a low cost and very good performance for applications that do not require communication distances of more than couple of tens of meters.

The interface with the external computer was operated through a USB connection, and data could be visualized online (see Figure 7b) and could be saved in the “.csv” format for further analyses.

#### 2.1.2. Train Unit with Distance Sensor

**Figure 8:**
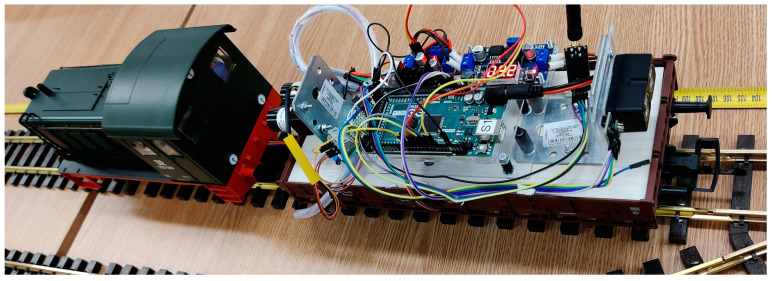
Mobile platform (SLAVE#1) equipped with LiDAR.

**Figure 9:**
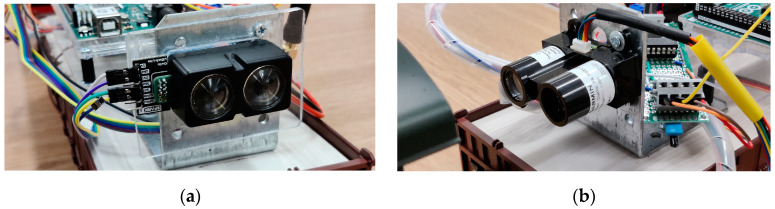
Two types of LiDAR sensors with different ranges: (**a**) 10 m LiDAR-Lite-V4, (**b**) 40 m LiDAR-Lite-V3.

The primary mobile train (SLAVE#1) (see Figure 8) unit was equipped with a LiDAR Lite V4 [28], see Figure 9a, with a maximum measurement distance of 10 m or a LiDAR LiteV3 [29], see Figure 9b, sensor that had a maximum measurement distance of 40 m.

The main controller was an ATmega2560 microprocessor that connected to the LiDAR sensor by the serial peripheral interface (SPI), which is a high-speed serial connection. The unit also had a radio nRF24L01 chip which was used to receive commands from the MCU control unit. The processor was also connected to a digital sensor that was used to determine the absolute speed of the train by measuring the pulses generated when a small magnet placed on the axel of the motor passed in front of a Hall sensor.

Furthermore, the processor was responsible for controlling the onboard DC–DC converter that controlled the speed of the train according to commands received over the radio network. The DC–DC converter had a variable pulse width modulation implemented by the L298N control circuit. The power for the entire unit was supplied from the rail the train ran on. For this application, a 20Vdc power supply was used to power the track.

#### 2.1.3. Train Unit without Distance Sensor

**Figure 10:**
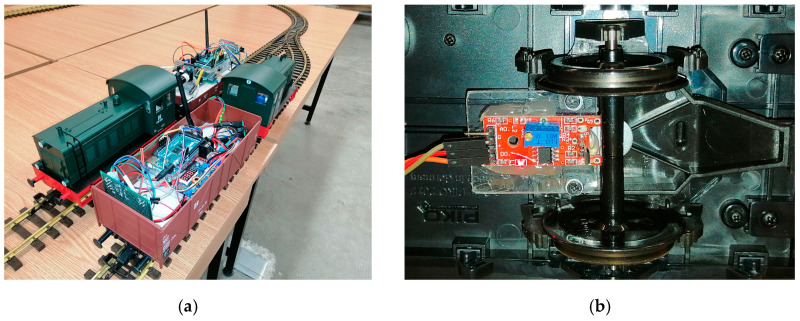
Experimental setup: (**a**) both mobile platforms—SLAVE#2 in the front and SLAVE#1 in the back; (**b**) speedometer with Hall sensor and magnet on the axle.

The secondary mobile train unit (SLAVE#2) was identical to the first train unit, with the exception of not being equipped with a LiDAR sensor. Both trains are presented in Figure 10a. It had the same microprocessor, the same radio module, the same Hall sensor (see Figure 10b) and the same DC–DC converter. Its main purpose was to act a variable speed target object that would be used to test the performance of the LiDAR sensor in terms of variable speed sensing capabilities.

### 2.2. Test Platform Software

The experimental model software was composed of functions; the implementation language was the *C* language and the compiler/linker tool chain was the *AVR-GCC* package [30].

#### 2.2.1. Communication Software

The communication software was responsible for the configuration and monitoring of the radio network. Each radio module was configured to have a unique identifier and a common radio channel. The modules communicated in the 2.4 GHz radio frequency, with this being made up of a number of channels; in our case, the channel used was number 95.

To actually send data over the radio network, a communication protocol was developed, which used data packets with the following configuration:

MESSAGE: SOF, LEN, OP, ID1, ID2, D1, D2, D3, D4, D5, SUM

where:

SOF (start-of-frame)—represented the start of a new message, it had the value 0x2A;

LEN—represented the length in bytes of the data package, its value was fixed at 0x05;

OP—represented the operation code;

ID1, ID2—represented the identifiers of the two mobile units, which were different from the radio ID’s, and identified the units inside the protocol;

D1 to D5—these were actual data bytes with sensed distance, absolute speed, relative speed, status and active pulse command;

SUM—represented the checksum of the message, which was used as an error detection method. If the sum of the package was not equal to the actual calculate sum, then the message was ignored. The checksum was calculated according to the following equation:

SUM = (ID1 + ID2 + D1 +D2 +D3 + D4 + D5) & 0xFF (9)

#### 2.2.2. Master Controller Software

The master control software was responsible for the management of the entire test platform. At the highest software level, it had two states when powering on:

- SETUP state—in which all the libraries, variables and constants of the program were defined; this was also where the hardware was configured;
- LOOP state—in which the actual functions of the program were implemented.

**Figure 11:**
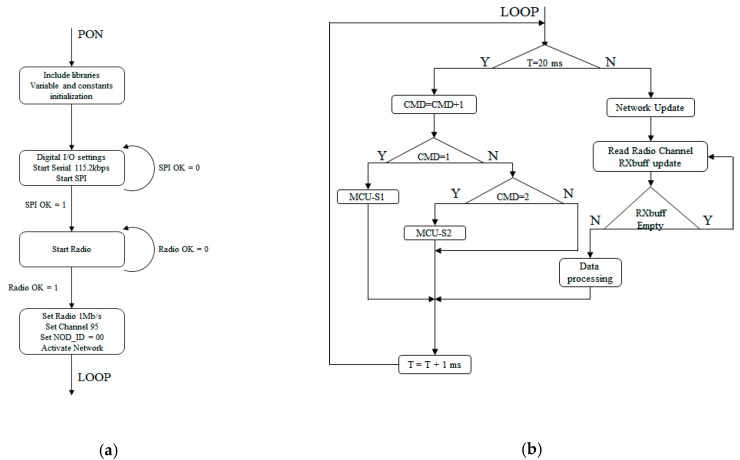
SETUP state (**a**) and LOOP state (**b**) diagrams for the MCU software.

A detailed diagram of the SETUP/LOOP states is presented in Figure 11:

The SETUP state permitted the definition of the necessary libraries, the declaration of all the variables used by the program, the initialization of the constants and the configuration of the hardware modules. The 4 digital lines, used to configure which secondary unit we were addressing and what type of motion we sent to it, were configured as digital inputs. The analog inputs were left at their default values—a reference voltage of 5 V and a resolution of 10 bits. This gave a precision of ~5 mV/bit. After the inputs/outputs were initialized, the serial connection was configured with a baud rate of 115.2 kbps. The radio communication module was set up by first initializing the SPI interface of the processor, if the SPI initialization failed, the program retried a couple of times before giving an initialization error. If it succeeded, it tried to send a start command to the radio module, and if this also failed, the program retried the start command before giving an error message. If the radio module returned an OK signal, the module was configured for channel 95, ID of 0x00 and 1 Mbps.

After the setup and configuration state, the software entered the LOOP state where all the functions were executed. The LOOP state was made up of two interdependent parts:

- Functions responsible for network monitoring, packet reading and updating;
- Functions responsible for unit control.

To be able to receive data from the two mobile units, the MCU software issued commands to the two mobile units and then monitored the radio network for responses from them. Upon receiving a data packet, the master software confirmed that the identifier and operation code were correct and updated the receive buffer with the requested data. After processing the data, the receiver buffer was cleared and the software went back to monitoring the network.

#### 2.2.3. Train Control Software

**Figure 12:**
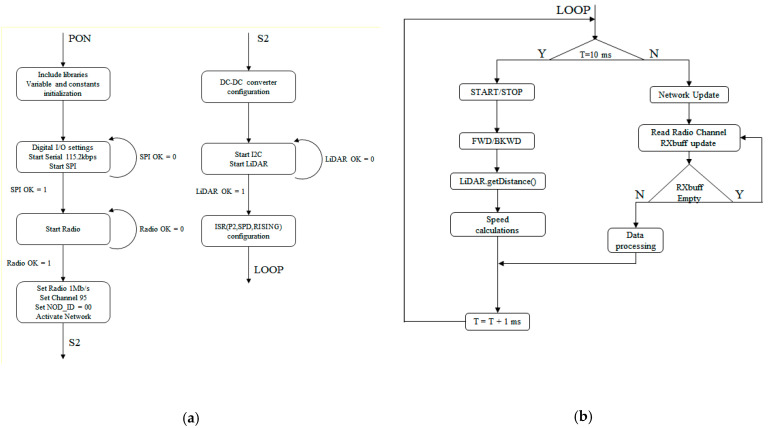
SETUP state (**a**) and LOOP state (**b**) diagrams for the SLAVE#1 and SLAVE#2 software.

Apart from the LiDAR-sensor-specific functions, the two mobile units had the same software implementation. The main diagram is presented in the following figure. It is similar to the MCU diagram with the difference that it had an additional state ISR ENCODER. This additional state was the interrupt service routine generated by the pulses from the Hall sensor. When a transition appeared on the input pin as a result of the magnet passing by the sensor, the processor entered the interrupt service routine (ISR) where the time between two consecutive pulses was measured, which was proportional to the absolute speed. The mobile unit software also had two states, SETUP and LOOP, but the implementation was different from the MCU, as seen in Figure 12.

With regard to the SETUP state, the first part was identical to the MCU, with the addition of the DC–DC converter module that had to be configured. The module was connected to pins 4 and 5 on the processor and its states were configured according to Table 3:

The LiDAR sensor also had to be configured; a start LiDAR command was sent to the sensor, and if this command failed, the processor would issue an error message.

The last configuration step was for the hall sensor encoder interrupt routine. This was accomplished via a function which took, as parameters, the pin on which the Hall sensor was connected, the type of signal to be monitored (high–low transition, low–high transition, or both) and the name of the ISR function. In this case, pin 2 was connected to the Hall sensor, the rising edge of the signal was counted and the function name was ”encoderSpeed()”.

The actual implementation of the ”encoderSpeed()” function is given below, in C-style code:

void encoderSpeed()

{

static unsigned long dt2 = 0; // static retains the value between function calls

dt1 = micros ();       // the time in us when the interrupt occurs

dt_us = dt1 - dt2;      // the time between two successive rising edges

dt2 = dt1;          // time update

}

**Figure 13:**
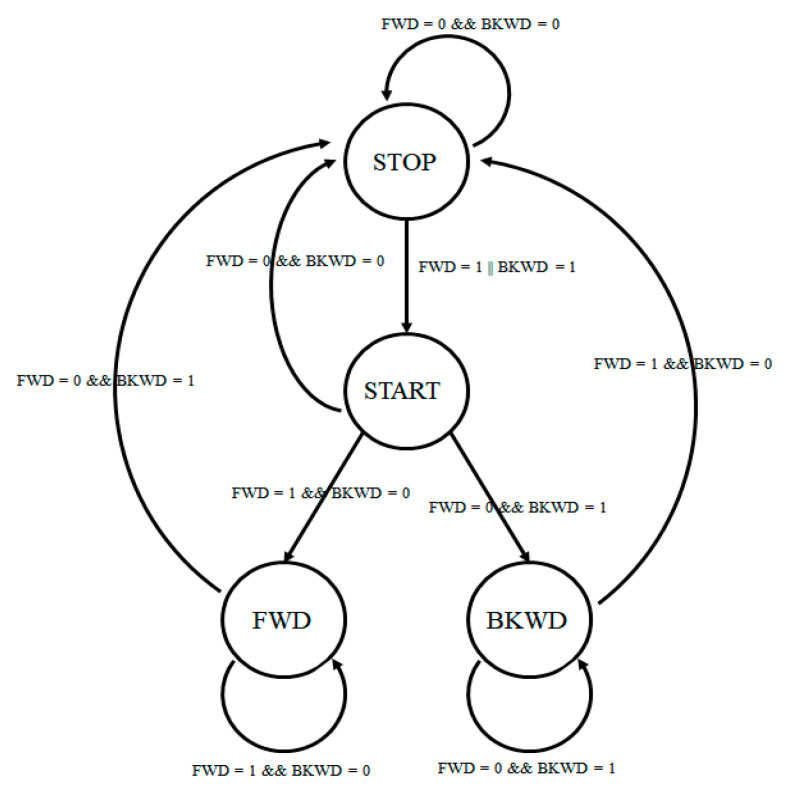
DC–DC converter control signal state diagram.

For the LOOP state, the radio network functions were identical to the master unit ones. However, new functions were implemented to handle the LiDAR measurements, the speed calculation and the control signal generation for the DC–DC converter. A time step of 10 milliseconds was set up to make it possible to execute the functions in a controlled manner. First, the START/STOP and FORWARD/BACK signals for the DC–DC converter were generated based on commands received from the MCU. The signals were processed by a state diagram, as shown in Figure 13, which assured correct implementation and the avoidance of erroneous signals.

The next function read the distance measurement from the LiDAR sensor; since this value was internally calculated by the sensor itself, all the program needed to do was to issue a ”LiDAR.getDistance()” command, which is a standard command from the library of the sensor. This measurement was scaled in centimeters and had a range of 0 to 1000 cm, corresponding to 0 to 10 m.

The last function was used to calculate the actual speed of the train from the time difference measured inside the interrupt function (ISR). Since the value of the time difference was in microseconds, the formula to obtain the speed was the following:

v = 10^6^/dt_us [rot/s] (10)

where dt_us is the name of the variable that holds the time difference. For performance reasons, the actual linear speed was calculated on the external computer, since it required floating-point calculations. The formula is given by Equation (8):

v_linear = V·π·D [m/s](11)

where D is the diameter of the wheel (35 mm).

#### 2.2.4. PC Software

**Figure 14:**
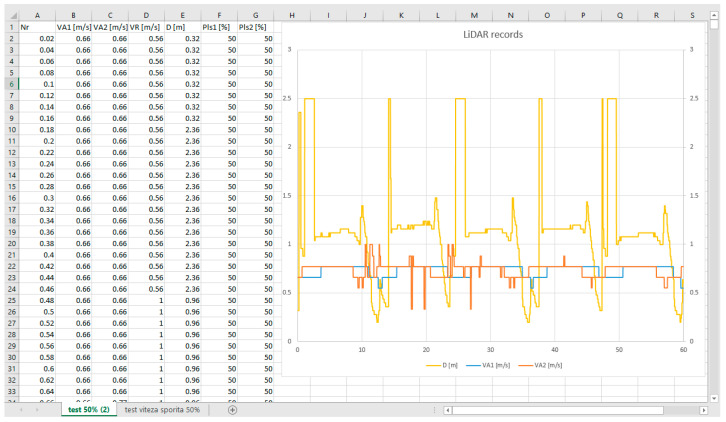
Data saved in csv file and plotted in Excel.

On the external computer, a program was installed that read the values the MCU send and plot’s and saved the data for further analysis; it was able to display a real-time graph of the distance measurement, numerical values for distance, both absolute and relative speeds, and the control signals, and it was also possible to save the data in a “.csv” file that could be viewed in Excel for further graphing, as seen in Figure 14.

The graph on Figure 14 presents, on the left side, the actual values of the reported distance and speed for both vehicles, and, in the right panel, the measured distance is shown (yellow graph). The SLAVE#1 followed SLAVE#2 on a 0-shape track, with the steady values related to the straight-line movement, while the transitions corresponded to objects around the laboratory detected when the train made 180° turns.

## 3. Results

### 3.1. Distance Measurement

With the SLAVE#1 vehicle placed on the track and doubled with a meter scale, we moved the vehicle in certain places, as described in row 1 from Table 4. The vehicle was moved by remote control, with the aim being to align, as much as possible, the frontal part of the LiDAR with the desired value. The value in row 2 represents the value measured on the meter scale, and the value in row 3 is the value that was transmitted by radio from SLAVE#1 to the MASTER and, consequently, observed on the software interface on the PC. In Figure 8, we can see the LiDAR and the metered scale observed near the track while the train was stopped at the 100cm mark. The measurements were performed with steady vehicle, with different light levels and with different colored targets at the end of the track.

The errors between the measured distance and the LiDAR-measured value were smaller than the 5% error of the device for the white target in daylight conditions.

In order to verify that the LiDAR detecting technique was not influenced by the color of the target, nor by the ambient light, additional tests were performed, and the results are presented in Table 5 and Table 6, the latter with the worst-case scenario for the brown target with low light (14 lux).

**Figure 15:**
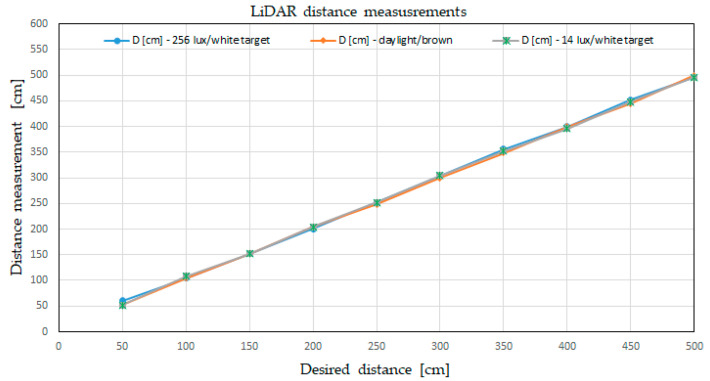
Plot of LiDAR distance measurements in various conditions

Once again, we can observe that the error was still less than the value of 5% provided by the producer of the LiDAR, and the measuring device was operating fairly with dark targets (low reflectivity) and at low ambient light. All three tests are presented in Figure 15, using the ”Desired distance” for the horizontal axis.

**Figure 16:**
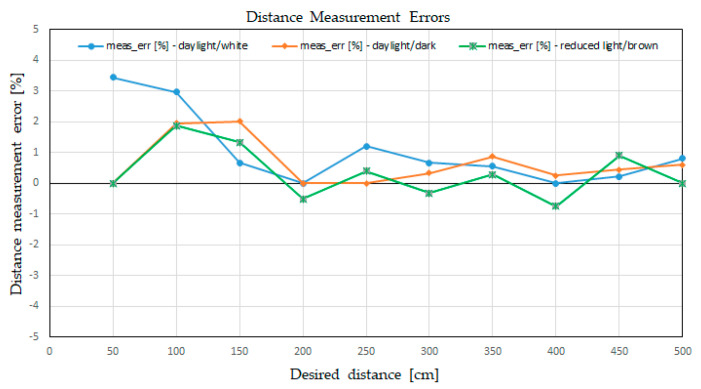
Plot of the distance measurement errors in various conditions.

The errors are presented in the Figure 16, where we can see that the LiDAR errors were mostly positives, meaning that the LiDAR was rounding the fractional values up to the centimeter level of thickness.

### 3.2. Speed Measurement

#### 3.2.1. Speed Measurement Using the on-Board Speed Sensor

The vehicle’s speed was measured using the on-board speed sensor. This was based on a one-pulse-per-rotation sensor consisting of one magnet placed on one free axle of the trailer and one position sensor placed on the bogie’s frame, as can be observed in Figure 10b. Placing a higher resolution sensor was not the aim of this experiment, and the speed of collection was reasonable for indoor testing.

The speed indication was provided by the controller using a timer for the on-state of the sensor’s input, and using a dedicated interruption for time counting. The time measured according to Equation (7) was sent to the master by radio communication, and the master performed the computation, as described in Equation (8).

To verify the speed measurement, we performed some tests in which we validated the measured speed compared with the calculated speed.

**Figure 17:**
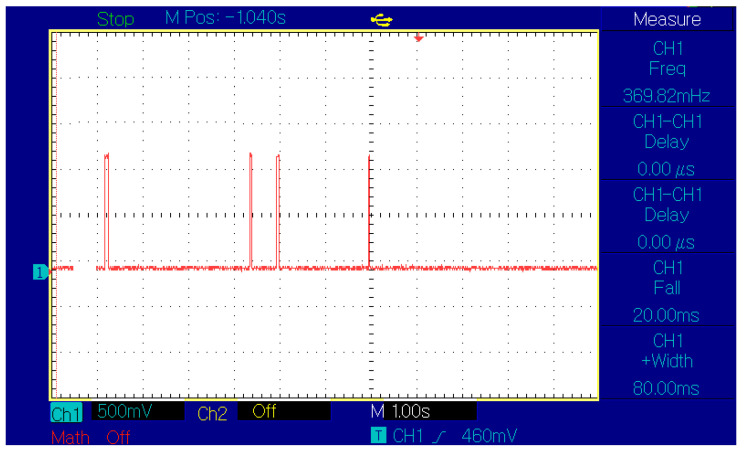
Speed indicator validation using an oscilloscope.

The initial time measurement was performed using an oscilloscope to monitor the signals from two sensors placed on the track at 2 m distance, connected to Ch1. In Figure 17, we can see a caption of the scope with first two spikes from SLAVE#2 running at a constant speed of 0.62 m/s and passing in 3.2 s, followed by the spikes generated by SLAVE#1 crossing the sensors in 2 s, representing an average speed of 1 m/s.

More precise tests were performed by dividing the 4-m-long distance by the chronometer time (s), and then measuring the speed in (m/s). With the train rolling at a constant speed, we performed three measurements in three successive trips and we used the average time to compute the speed, as presented in Equation (12) and in Table 7.

v_a_ = d/t_av_·(m/s) (12)

The speed reported by the trains was verified using the distance-divided-by-time method.

#### 3.2.2. Speed Measurement Using the Reported Distance

**Figure 18:**
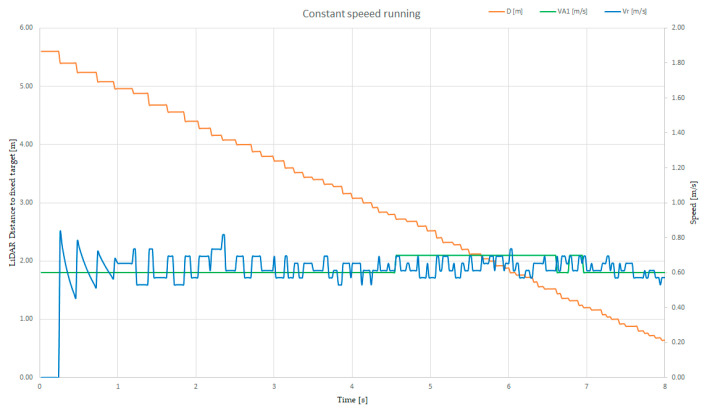
Plot of the distance (orange), relative speed (blue) and actual speed (green) of the tram with a LiDAR sensor, at constant running speed.

Using a validation technique that involves reading the distance from the reported and recorded parameters of a train directed to a fixed target, one can find the correspondence between the speed computed from the LiDAR sensor measurement and the value provided by the on-board speed sensor. In Figure 18, we present a graph based on the data recorded from the PC while the SLAVE#1 vehicle ran on the O-shape line toward a fixed target.

We controlled the speed of the vehicle to ensure that it was constant; in this case, the speed was 0.6 m/s (the green graph on the secondary vertical axis), while the distance was represented with orange line, descending from 5.6 m down to 0.6 m. The relative speed calculated from the distance, denoted as v_r_ (blue line scaled on the secondary vertical axis), was calculated using validated data (data recorded during the U-turn was not valid, and thus, we did not consider it in this application) and it represented the trip divided by the time interval. In this graph, the time interval was 1s.

Because the distance was measured and reported at different time slots, the recorded distance information had unequal steps, with this being visible while computing the relative speed information. Compared with the measured speed (green line in Figure 18), the values of the computed speed were in closer proximity. This issue had to be solved using software methods in the latter stages of the project, with the aim of reaching an error rate of less than 5% in terms of relative computation speed based on the distance measurement.

### 3.3. Relative Speed Measurement

While the vehicle speed was measured using the on-board speed sensor, the relative speed was computed from the distance difference between two successive measurements, divided by the time between those two measurements.

**Figure 19:**
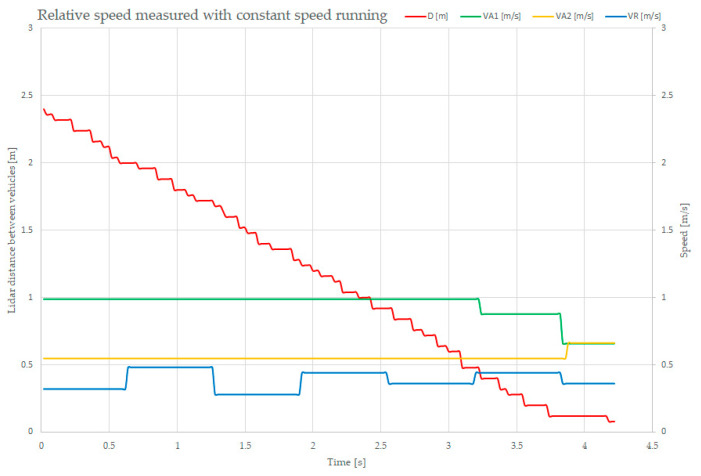
Plot of the relative distance and speed measurement at constant running speeds.

The test consisted of placing the two vehicles on the same track, with each of them operated at constant (but different) speeds, to record the distance between them, their times, and both of their sensed speeds. The results of the experiments are shown in Figure 19.

The red-lined graph is the measured and reported distance between the two vehicles, descending from 2.4 m down to 0.1 m, meaning that the two vehicles collided. The green-lined graph shows the on-board measurements of the SLAVE#1 speed (1 m/s), and the yellow one represents the on-board measurements of the SLAVE#2 speed (0.66 m/s). The blue speed is the computed and reported relative speed between the two vehicles. For stability reasons, the speed is integrated over a 0.6 s time slot. In order to improve this speed measurement, some software-based refinement procedures must be implemented.

The results of these measurements are important as they were recorded based on the sensed distance between the two vehicles, computed using the sensor, as described before. The relative speed, which was computed using the average distance values, could be more precise.

## 4. Discussion

The starting point for our research was the fact that the most dangerous situation regarding tram safety is when a tram is running at high speed on dedicated straight lines. The braking distance can vary, at a speed of 50 km/h, from 94 m using a service brake, down to 50 m when using emergency braking with sanding, with these values being computed according to the European standard regarding braking systems for trams (EN 13452). For every second of delayed braking when running at 50 km/h, the braking distance is extended by 14 m, thus increasing the possibility of a collision with another tram located to the front if the driver is unable to react in time.

We intend to develop a tramcar collision detection system, using LiDAR sensors, which will measure the distance between one tramcar and another tramcar in front of it and, based on that information as well as the absolute speed of the tramcar and its speed relative to the tramcar in front, issue an early warning to the driver when that distance is below the safe braking distance. The alternate solutions for measuring the time-to-impact, which are used in the car industry, namely FCW and EBS, are not efficient in tram applications.

The LiDAR sensors can have long-range angle with a narrow field-of-view (FOV) or a wide FOV with a short range. The car industry uses the wide FOV LiDAR, based on better braking capabilities. For the tram, we can use narrow FOV to preventing collisions, especially on straight and separated lines. The LiDAR used in the experimental model has a 10m range and a FOV of 4.77°.

LiDAR sensors have good responses at important distances (with this being the objective of the outdoor tests) and small speed errors (at medium speed), and thus, they are good enough for safety distance estimations. Upscaling the LiDAR system for tram detection with a 100 m sensing range will be the objective of the project’s next stage.

The current implementation aims mostly at validating the distance and the relative speed measurements using LiDAR devices, as well as testing relevant software procedures to improve the precision and the repeatability of their measuring capacities. Using basic sensors can provide rough data for future developments in both hardware and software.

With the test platform being a down-scale model for indoor use, the sensor’s accuracy was a concern for us, and while the LiDAR measurements were in the range specified by the datasheet, the Hall sensor used for the absolute speed measurements had a low accuracy because of its poor resolution.

Furthermore, the method of reading the distance from the reported and recorded parameters of a train, directed to a fixed target, led to a correspondence between the speed computed by the speed sensor and the value provided by the distance sensor.

The relative speed computed became more precise when using longer measuring intervals. It was found that it is possible to obtain very precise values with some delay, or to obtain fast information with less precision. This software-refining optimization will be the focus of the final stage of our project. The end goal of the project is to design an early warning collision detection system for tramcars using LiDAR sensors.

Some sensors can provide the relative speed value as a readable variable available through the serial interface I2C. Once again, in this work, we were focused on revealing the rough data and finding the solution to convert them to stable, repetitive and usable variables for the electronic control unit.

## 5. Conclusions

The distance and relative speed sensor we implemented for this experimental vehicle model is a precise measuring device.

The sensor used has a range of 10 m; indoor experiments were performed on an O-shaped track, which allowed a direct trip of 5.4 m. This value was used for distance validation with different colored targets and with various degrees of illumination, demonstrating that the LiDAR, using its own light emission, is an optical measuring device that is insensible to the environment.

Our contribution was the development of an experimental model for indoor dynamic tests for long-range LiDAR, starting from two regular scale models of trains, by inserting a speed control device into the trailer wagon and by controlling it using a 2.4 GHz radio network from a stationary controller that acted as an independent remote control for both trains. In this system, one of the trains carried the LiDAR device that could be used to measure the relative distance and the relative speed. Both trains used a radio network to provide online information about their sensed distance, speed and status. Data were stored and observed online on a PC connected to the remote unit. A software program called TRAMDAR was developed especially for this.

The results consist of the validation of the measurement methods, especially the software-based measurements, using data recorded during extensive tests.

Future work will aim at implementing this measurement technique on a full-scale model using the core software developed for the indoor model and a 180-m-range LiDAR acting as an advanced Frontal Collision Warning system and as part of a dedicated Emergency Braking System for trams.

## Figures and Tables

**Table 1:** Examples of methods used to determine distance.

Type of Distance Measurement Method			
Optical methods	Passive	Geometrical	-
	Active	Geometrical	-
		Time-of-flight	Direct methods
			Indirect methods
		Interferometry	-
Non optical methods	-		

**Table 2:** Examples of LiDAR uses in vehicle safety [11].

Level of Autonomy	Example of Applications	LiDAR Adoption
Level 1	Automatic Emergency Braking (AEB) Adaptive Cruise Control (ACC) Lane Keep Assist (LKA)	Little or no LiDAR
Level 2	Parking Assist (PA)) Traffic Jam Assist (TJA)	Some will use LiDAR
Level 3	Highway pilot	Most will use LiDAR
Level 4	Automated Urban Mobility	LiDAR is necessary
Level 5	Full Automation	LiDAR is necessary

**Table 3:** DC–DC converter state based on pin values.

Pin 4	Pin 5	State
0	0	Stop
0	1	Forward
1	0	Backward
1	1	Stop

**Table 4:** Distance measurements with ambient light intensity of 265 lux (daylight) and a white target.

**Desired**	(cm)	50	100	150	200	250	300	350	400	450	500
**Dmas**	(cm)	58	101	151	200	249	302	354	400	451	492
**DLiDAR**	(cm)	60	104	152	200	252	304	356	400	452	496
**LiDAR Error**	(%)	3.44	2.97	0.66	0	1.2	0.67	0.56	0	0.22	0.81

**Table 5:** Distance measurements with ambient light intensity 265 lux (daylight) and a dark target.

**Desired**	(cm)	50	100	150	200	250	300	350	400	450	500
**Dmas**	(cm)	52	102	149	204	248	299	345	399	442	497
**DLiDAR**	(cm)	52	104	152	204	248	300	348	400	444	500
**LiDAR Error**	(%)	0	1.96	2.01	0	0	0.34	0.87	0.25	0.45	0.6

**Table 6:** Distance measurements with ambient light intensity 14 lux (twilight) and a dark target.

**Desired**	(cm)	50	100	150	200	250	300	350	400	450	500
**Dmas**	(cm)	52	106	150	205	251	305	351	399	444	496
**DLiDAR**	(cm)	52	108	152	204	252	304	352	396	448	496
**LiDAR Error**	(%)	0	1.88	1.33	−0.5	0.39	−0.32	0.28	−0.75	0.9	0

**Table 7:** Speed measurement with ambient light intensity of 265 lux and white target.

**v_s_ Average speed** **(From mobile platform)**	(m/s)	0.99	0.88	0.66	0.22
**d Timing distance**	(cm)	400	400	400	400
**Timing t_1_**	(s)	4.08	4.58	6.06	15.04
**Timing t_2_**	(s)	4.18	4.62	6.05	14.72
**Timing t_3_**	(s)	4.12	4.63	6.17	16.62
**Average time t_av_**	[s]	4.1264	4.61	6.093	15.46
**v_a_ Speed (average time)**	(m/s)	0.969	0.867	0.656	0.258
**Speed error**	(%)	2.12	1.47	0.61	17.6

## Data Availability

Not applicable.
